# Supplementary material for: Political context of the European vaccine debate on Twitter
Source: Sci Rep. 2024 Feb 22;14:4397. doi: 10.1038/s41598-024-54863-7 (PMC10883931; doi:10.1038/s41598-024-54863-7)
Supplement: Supplementary file 1 — Supplementary Information. [file 41598_2024_54863_MOESM1_ESM.pdf]

Political Context of the European Vaccine Debate on Twitter

Giordano Paoletti[1,2], Lorenzo Dall'Amico[1], Kyriaki Kalimeri[1], Jacopo Lenti[3,4], Yelena Mejova[1,*], Daniela Paolotti[1], Michele Starnini[3,5], and Michele Tizzani[1]

[1]ISI Foundation, Turin, Italy
[2]Department of Control and Computer Engineering, Politecnico di Torino, 10129 Turin, Italy
[3]CENTAI, Turin, Italy
[4]Department of Computer, Control, and Management Engineering Antonio Ruberti, Sapienza University of Rome, Rome, Italy
[5]Departament de Física, Universitat Politècnica de Catalunya, Campus Nord, 08034 Barcelona, Spain
[*]yelenamejova@acm.org

Supplementary Information

1 Data Volume

The volume of the data collected can be seen in the Figure S1. We can observe that the volume increases drastically during COVID-19 (note the logarithmic scale on the y axis). The missing time frame between periods 1 and 2 encompasses the very start of the COVID-19 worldwide epidemic. Unfortunately, it coincides with a technical difficulty in collecting the increased volume of data. Due to the highly speculative and uncertain nature of that time period, we also exclude it in our analysis of the vaccination debate.

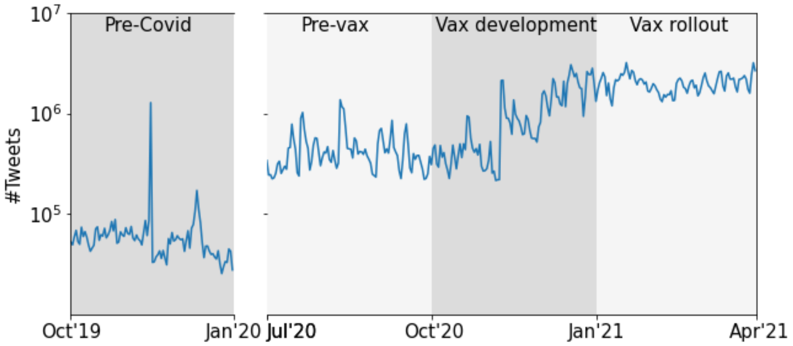

Figure 1. Number of tweets over time, four periods identified by background color.

2 Evaluation of Geo-localization Quality

Our approach of geo-locating users using the Location field of their self-description suffers from several drawbacks, including people lying, writing in non-locations, or having ambiguous or homonymous locations. Thus, we estimate the accuracy of our geolocation method by comparing it to the information shared by the users that includes the precise geo-coordinates of their location. We consider such geo-location for users using the official languages of the country (ones that we have selected for this study), or other languages (ones we did not consider). The list of official languages can be found in Table S2. As Table S1 shows, the accuracy for the official languages ranges around 0.95. The countries with lowest accuracy are Portugal (0.73) and Austria (0.88). Note that the accuracy for the other languages is much lower, justifying our selection of only those posts which use the official languages of the country.

3 RT-Networks Sizes

Table S2 shows the official languages for each selected country, as well as the number of users in the Giant Weakly Connected Component of the RT Networks for each time period. We apply a threshold of 300 users to the networks, which excludes 7

Table 1. Accuracy of user profile-based geolocation, compared to the GPS coordinates of the post, by official languages of the country and other languages. Instances where fewer than 10 users are available are left blank.

	Official lang.		Other lang.	
**Country**	**# Users**	**Accuracy**	**# Users**	**Accuracy**
**AT**	34	0.882353	14	0.500000
**BE**	57	0.947368	12	0.750000
**CH**	32	0.843750	15	0.466667
**CZ**	7		9	
**DE**	309	0.964401	54	0.555556
**DK**	24	1.000000	3	
**ES**	525	0.927619	53	0.471698
**FI**	27	0.962963	5	
**FR**	182	0.939560	62	0.209677
**GB**	582	0.903780	93	0.483871
**GR**	19	0.947368	8	
**IE**	47	0.914894	14	0.357143
**IT**	486	0.967078	131	0.236641
**NL**	97	0.969072	26	0.576923
**PL**	70	1.000000	17	0.764706
**PT**	100	0.730000	2	
**SE**	79	1.000000	12	0.666667

networks in the first period, as signified by the lack of numbers in the table.

Table 2. Number of nodes in the Giant Weakly Connected Component of the RT-Network of each country/period.

	**Period**	**1**	**2**	**3**	**4**
**country**	**Official lang.**	**# Users in the GCC**			
AT	de		1159	3825	6970
BE	nl, fr, de		1225	5037	9547
CH	de, fr, it		837	2727	4774
CZ	cs		535	2007	3778
DE	de	5527	11771	32337	58472
DK	da		468	1496	2625
ES	es	26634	100284	153274	216996
FI	fi, sv		905	2114	4242
FR	fr	17043	34352	92912	148523
GB	en	24333	98772	269185	392974
GR	el	790	3331	6461	6657
IE	en	2850	5498	19209	34053
IT	it	5514	12819	31979	50169
NL	nl	1425	5042	13940	20373
PL	pl	1874	5252	9356	12377
PT	pt		7482	17682	15598
SE	sv	654	730	2760	5426

4 VHE Score Robustness

We check whether the VHE score median and mean correlate with the number of users in the country, and find no such correlation. However, VHE score standard deviation has a positive correlation (0.648) with the number of users. This is expected, because the larger the network, the larger the number of communities that the CD algorithm can find.

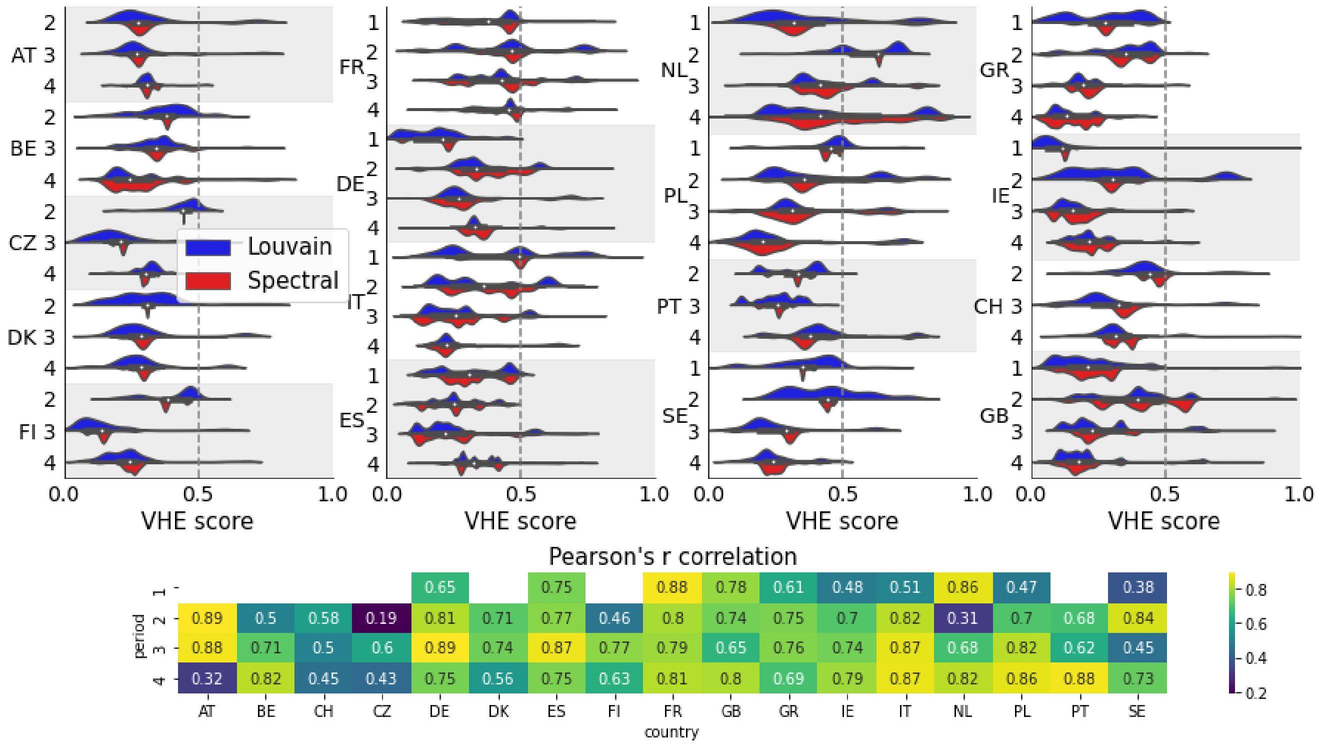

Figure 2. Comparison of the VHE scores computed using Louvain and Spectral Clustering as community detection algorithm. White cells in the heatmap indicate country/periods having fewer than 300 users, while all the Pearson's r are significant at $p < 10^{-5}$.

We also test the robustness of the VHE score assignment to the choice of community detection algorithm. We repeat the computation of users' VHE scores using Louvain algorithm, and the manually annotated tweets used in the paper (sampled using Spectral Clustering stratification). As can be seen from the Figure S2, the distributions' shape obtained with Louvain resembles that of Spectral Clustering. Notably, there is a strong linear relationship between the two scores, as expressed by Person's r shown in the heatmap (a median of r=0.741). The difference becomes more pronounced for smaller networks. In general, a higher variance in the scores' distribution is expected, since this method looks for partitions with a high number of communities, without, moreover, giving the possibility of establishing an upper-bound on this number, like that of $k = 15$ which we imposed to Spectral Clustering for interpretation purposes. The ability to choose the number of communities also allowed us to perform a stratified sampling of the tweets for annotation, which would have been difficult with Louvain's output. Given the results above, since to answer our RQs we use linear regression and correlations, the results would likely be similar if Louvain algorithm was used instead.

5 Results with Louvain-based VHE score

To make sure the results are robust to the selection of the clustering algorithm, we computed the main results for Research Questions 1 & 2, as can be seen in Figures S3-S6. Overall, the main findings do not change, compared to those shown in the main manuscript which use Spectral Clustering. Those most affected by the selection of the algorithm are the least significant results in Figure S6, however they do not change the main result of the RQ, mainly that there are few countries having a strong bias in terms of politicization and exposure to vaccine-hesitant content.

6 VHE score validation

To validate the computation of Vaccine Hesitancy Endorsement score per user, we perform a manual annotation exercise. For four of the largest countries in our dataset, Italy, France, Spain, and the United Kingdom, we stratify the VHE score into terciles. From each tericle we sample 15 users that have posted (or retweeted) at least 5 tweets, and annotate a sample of 5 tweets from each. We annotated a total of 900 tweets, and computed the difference between anti- and pro-vax tweets for each user and correlated it to their VHE score. As for one country, we found only 3 anti-vax tweets, we sampled further 5 users from the first

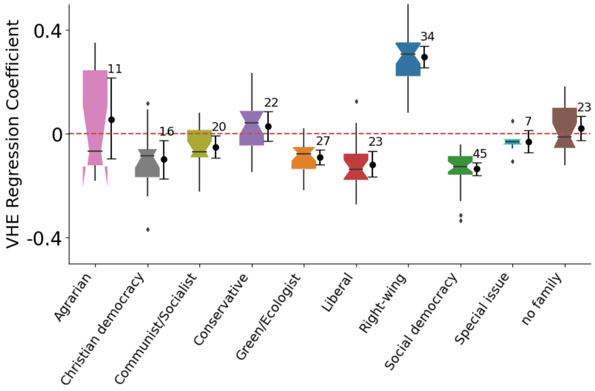

Figure 3. Distribution (boxplots) of OLS coefficients modeling users' VHE score by their interest in parties, grouped by families using ParlGov. Accompanying points and whiskers indicate a 99% bootstrapped confidence interval. Numbers indicate how many parties are in each group.

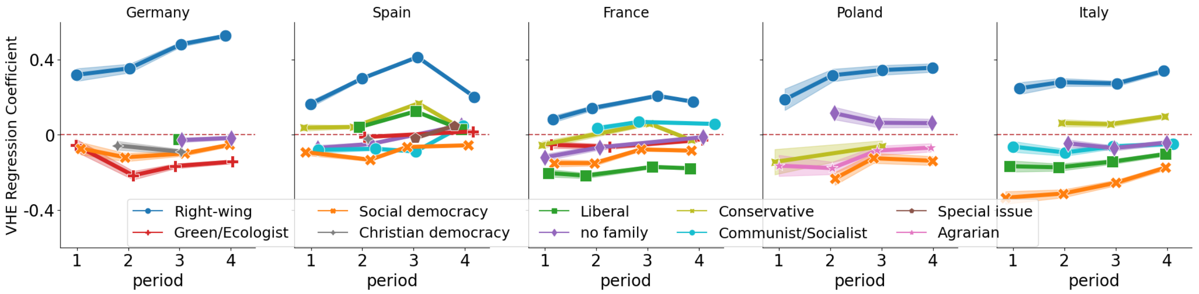

Figure 4. Significant OLS coefficients (at $p < 0.01$ with Bonferroni correction) for user interest in parties. grouped in families using ParlGoV, and their 99% confidence intervals. Showing countries having sufficient model fit over 4 time periods.

and fifth quintiles. The summary of the annotation and the Spearman correlation with the VHE scores can be seen in Figure S7. The correlations are 0.56 ($p < 0.001$) for Italy, 0.55 ($p < 0.001$) for France, 0.38 ($p < 0.01$) for Spain and 0.38 ($p < 0.01$) for the United Kingdom. As can be seen from the figure, out of all anti-vax tweets, 50-83% were for users having VHE score in the highest tercile. We conclude that the proposed approach is a good proxy for the stated endorsement of the no-vax stance.

7 Details of Linear Regression

In this section, we offer a concise overview of the implementation specifics for the Ordinary Least Squares (OLS) fit. This encompasses the variables under consideration, starting from the presentation of the fitting equation, which can be decomposed as follows:

$$VHE^{(u)} \sim \beta_0 + \sum_{i=1}^{K} \beta_i X_i^{(u)} + \sum_{j=K+1}^{N} \beta_j Y_j^{(u)}$$

Where

1. β represents the weights to be fitted,
2. $X^{(u)} = \{X_i^{(u)}\}_{i=1}^{k}$ is the set of confounding variables, and this feature set remains constant across the various fits. The confounding variables include: the number of followers and followees, daily posting rate, weighted in-degree and weighted out-degree (number of retweets they had, and number of retweets they made in the vaccine debate, respectively), and the proportion of followed users who are politicians (political interest, defined above).
3. $Y^{(u)} = \{Y_j^{(u)}\}_{j=K+1}^{J}$ is the set of features describing the political interest of the user u. Initially, for each country, this set is unique and comprises the fraction of politicians followed for each party in the dataset. The complete list of parties for each country is available at the following link: https://github.com/GiordanoPaoletti/

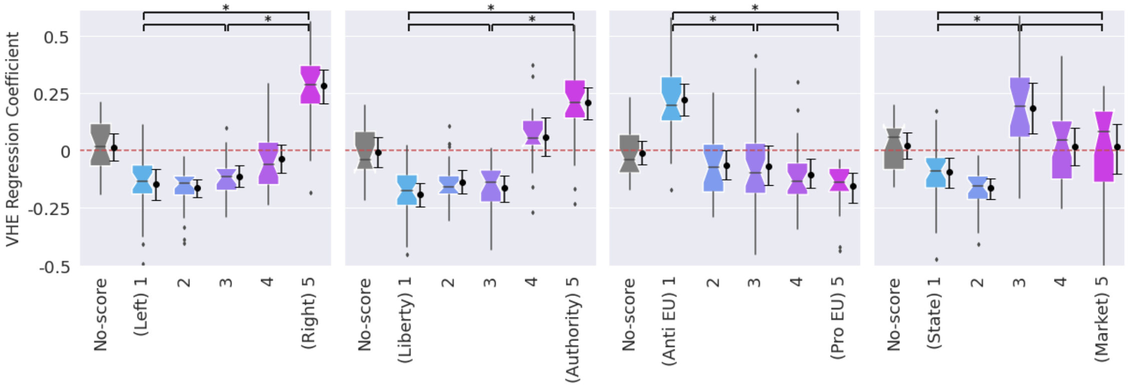

Figure 5. Distribution (boxplots) of OLS coefficients modeling users' VHE score by their interest in parties having one of four dimensions defined by ParlGov, grouped in quintiles. Accompanying points and whiskers indicate a 99% bootstrapped confidence interval. Horizontal brackets on top indicate the comparisons among quintiles 1, 3 and 5, * signifies whether one distribution is statistically greater than the other (one-sided Mann-Whitney U test at $p < 0.01$ with Bonferroni correction).

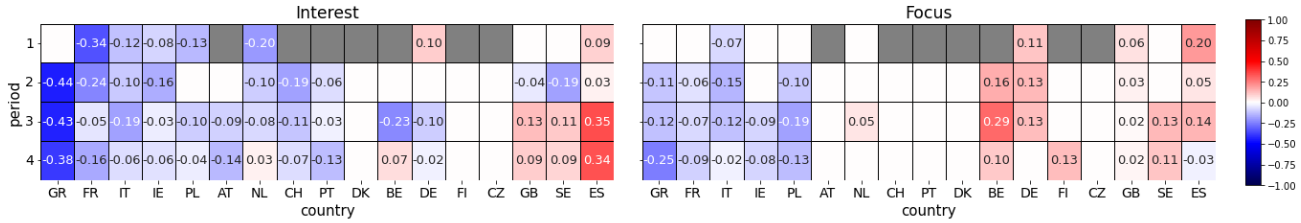

Figure 6. Spearman correlation between political interest and VHE score (left) and political focus and VHE score (right) (see RQ2 Methods). Grey cells indicate country/periods having fewer than 300 users. White cells indicate a non-significant correlation.

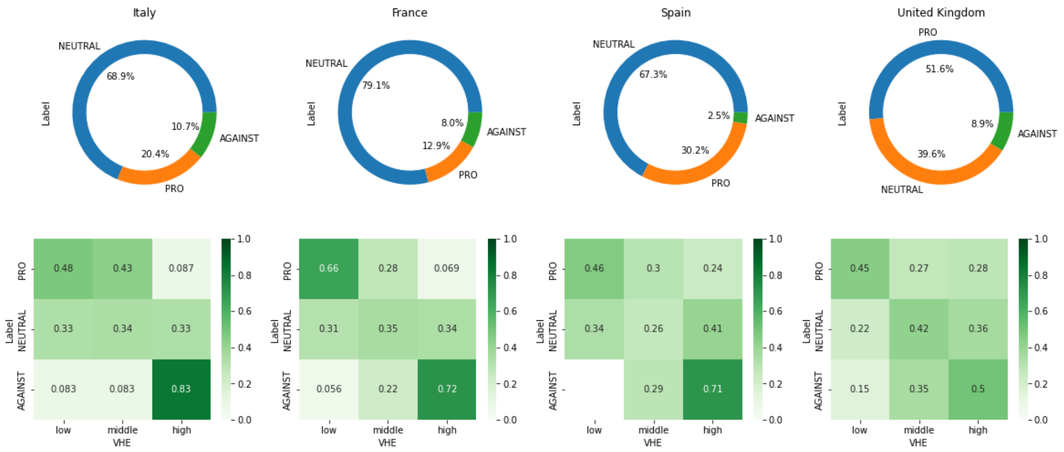

Figure 7. Summary of annotation results validating the VHE score. Ring plots show the percentage distribution of tweets per label, while heatmaps illustrate how these percentages are distributed among the terciles of VHE score distribution.

Political-Issue-or-Public-Health. For example, when modeling the VHE scores of users in Germany in a particular period, parties considered are *Alliance 90 / Greens*, *Alternative for Germany*, *Christian Democratic Union / Christian Social Union*, *Free Democratic Party*, *Other*, *PDS - The Left*, *Social Democratic Party of Germany*, *independent politician*.

For Figures 2 and 3, we group the political parties by party family, as indicated by ParlGov. The resulting list of fitting variables is: number_of_followers, daily_posting_rate, weighted_in_degree, political_interest, *Conservative*, *Social democracy*,

Green/Ecologist, Liberal, *Christian democracy*, *Communist/Socialist*, *Agrarian*, *no family*, *Right-wing*, *Special issue*.

For Figure 4, we group the political parties by quintiles in each of the four dimensions specified by ParlGov. This would result in four regressions, one for each dimension: left-right, liberty-authority, anti - pro EU, and state-market. So, keeping the confounding variables X unchanged, we define a set of 6 features Y (i.e. one for each quintile and the sixth to group parties with an unknown score).

Figures S8 - S19 show the scatterplots representing the performance of the regression models described above. In each, the x axis is the true VHE score of the user and the y axis is the model's prediction. Shown are only the regressions in which Adj $R^2 \geq 0.1$ (same threshold is used in the paper for analysis). Note that the score extends beyond the 0 to 1 range because we standardize the target variable to make a consistent comparison among OLS weights across countries and periods.

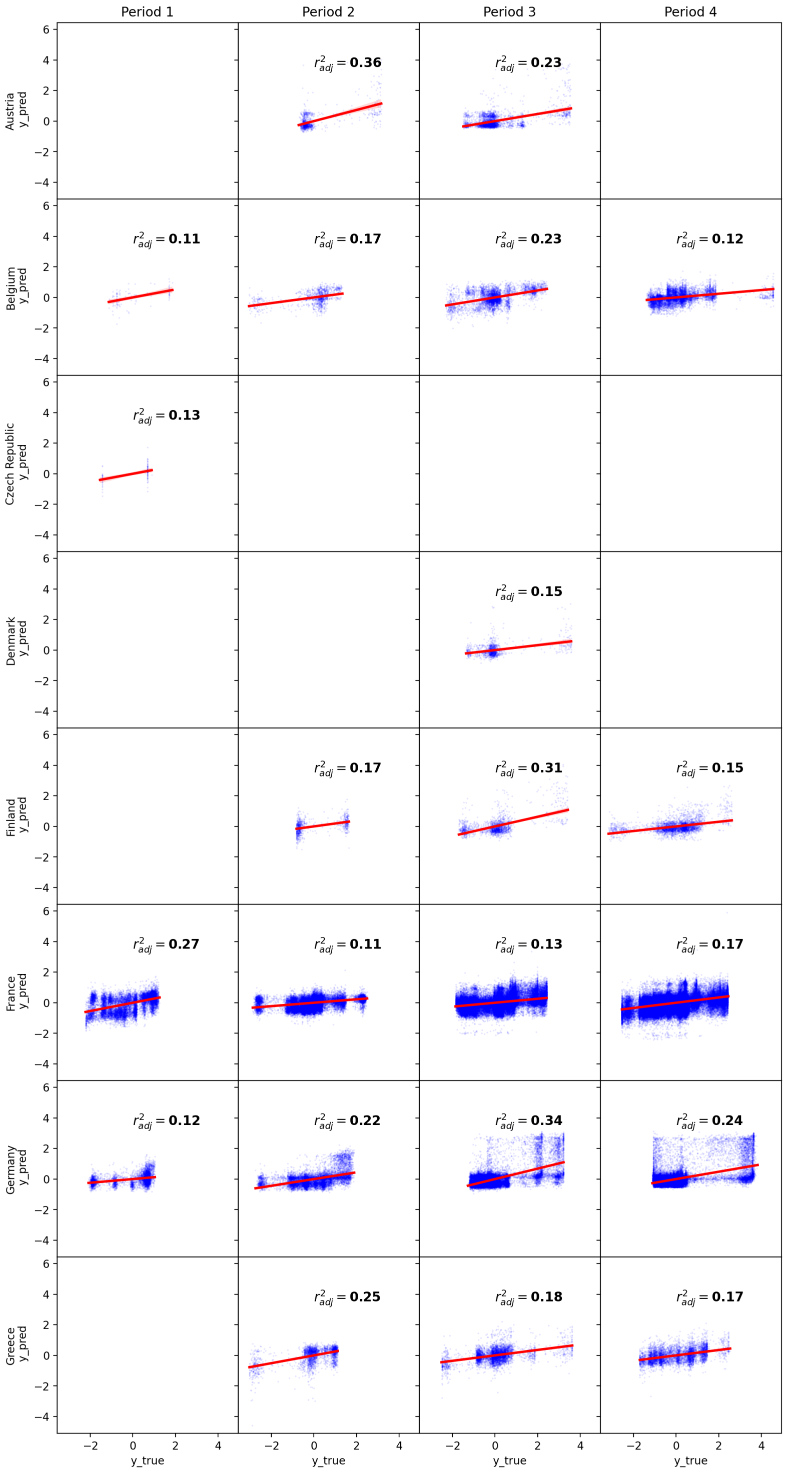

Figure 8. Regression performance modeling VHE score by parties (part 1). The x-axes represent the real target y_{true} (user's VHE score) and the y-axis the predictions from OLS models (y_{pred}).

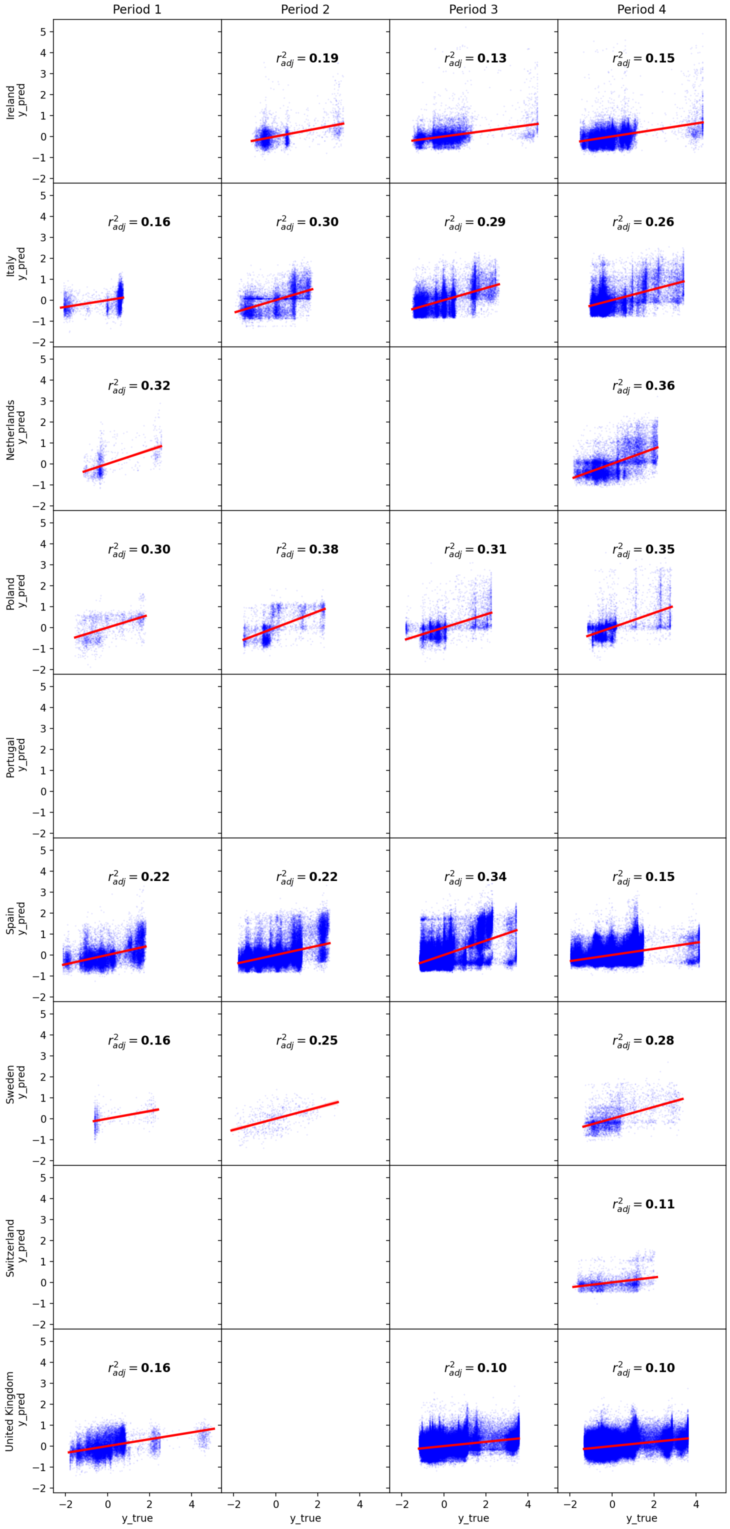

Figure 9. Regression performance modeling VHE score by parties (part 2). The x-axes represent the real target y_{true} (user's VHE score) and the y-axis the predictions from OLS models (y_{pred})

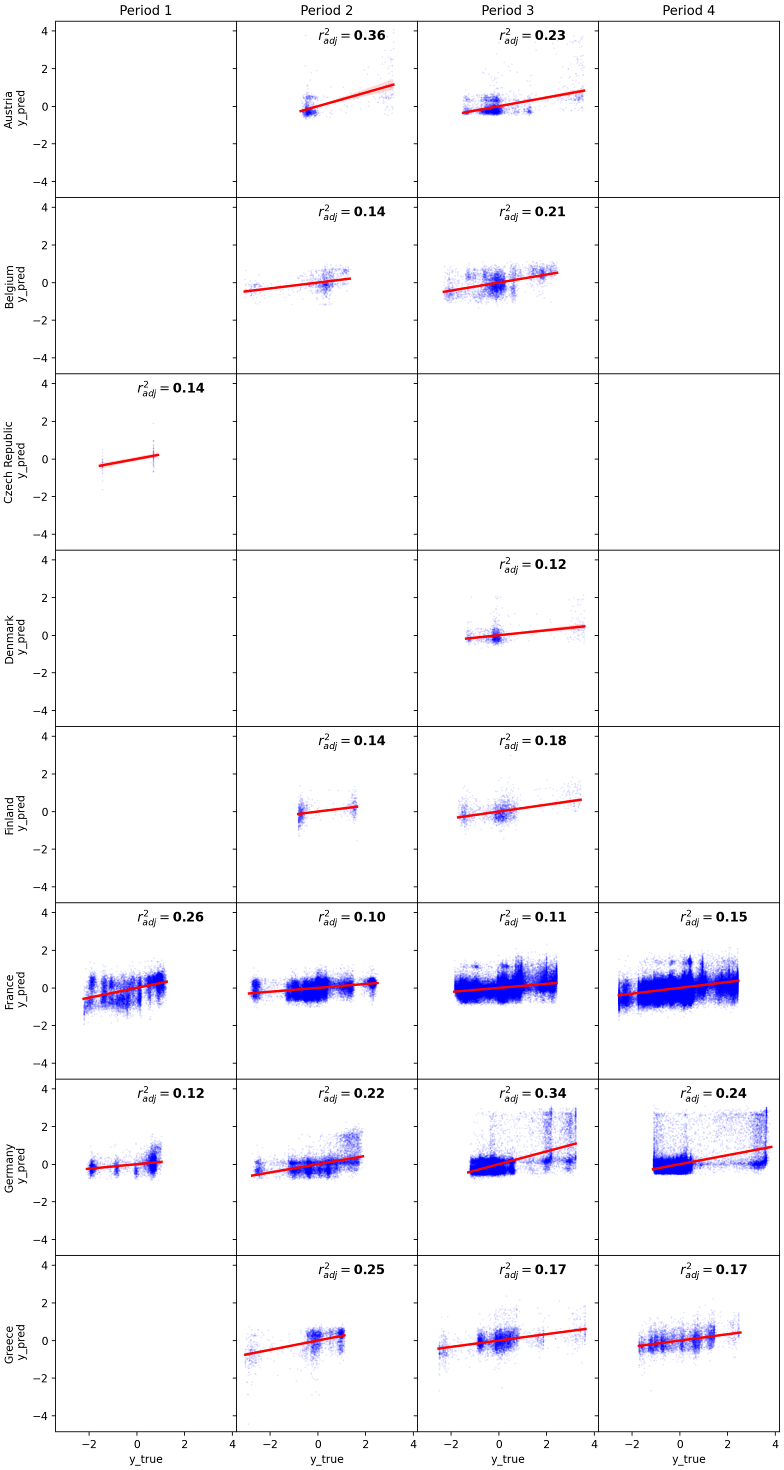

Figure 10. Regression performance modeling VHE score by parties grouped by families using ParlGov, (part 1). The x-axes represent the real target y_{true} (user's VHE score) and the y-axis the predictions from OLS models (y_{pred}).

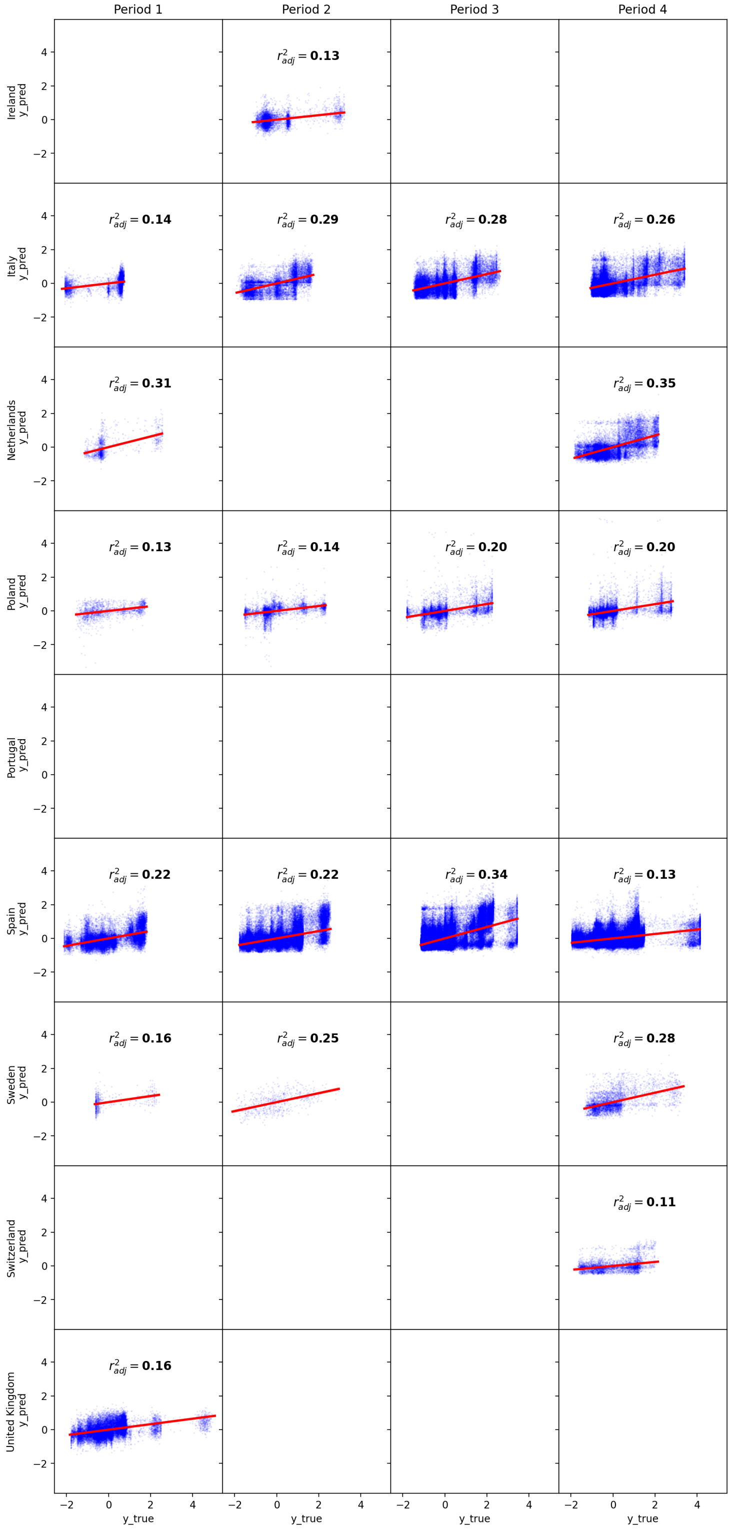

Figure 11. Regression performance modeling VHE score by parties grouped by families using ParlGov, (part 2). The x-axes represent the real target y_{true} (user's VHE score) and the y-axis the predictions from OLS models (y_{pred}).

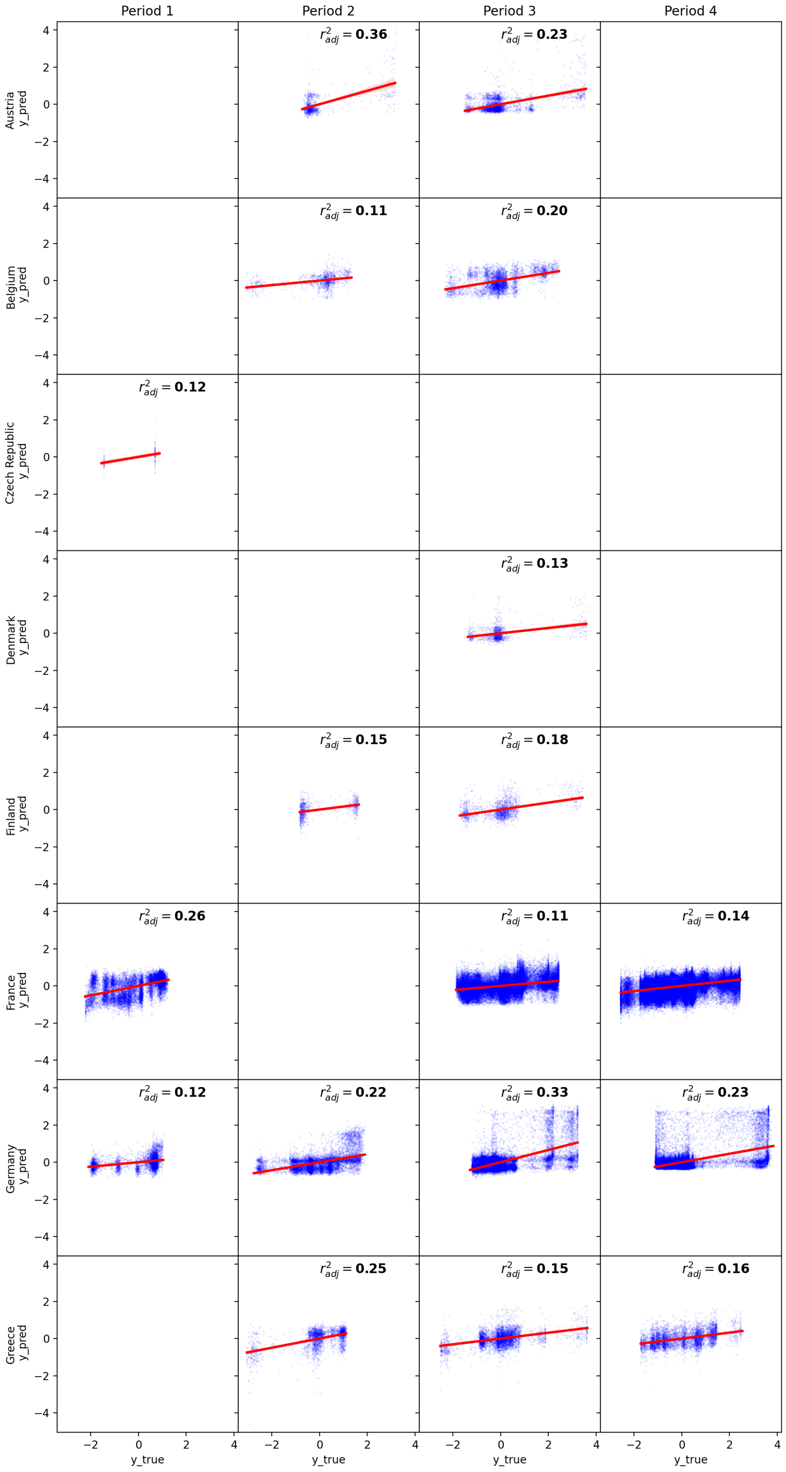

Figure 12. Regression performance modeling VHE score by parties grouped by quintiles of the left-right dimension specified by ParlGov (part 1). The x-axes represent the real target y_{true} (user's VHE score) and the y-axis the predictions from OLS models (y_{pred}).

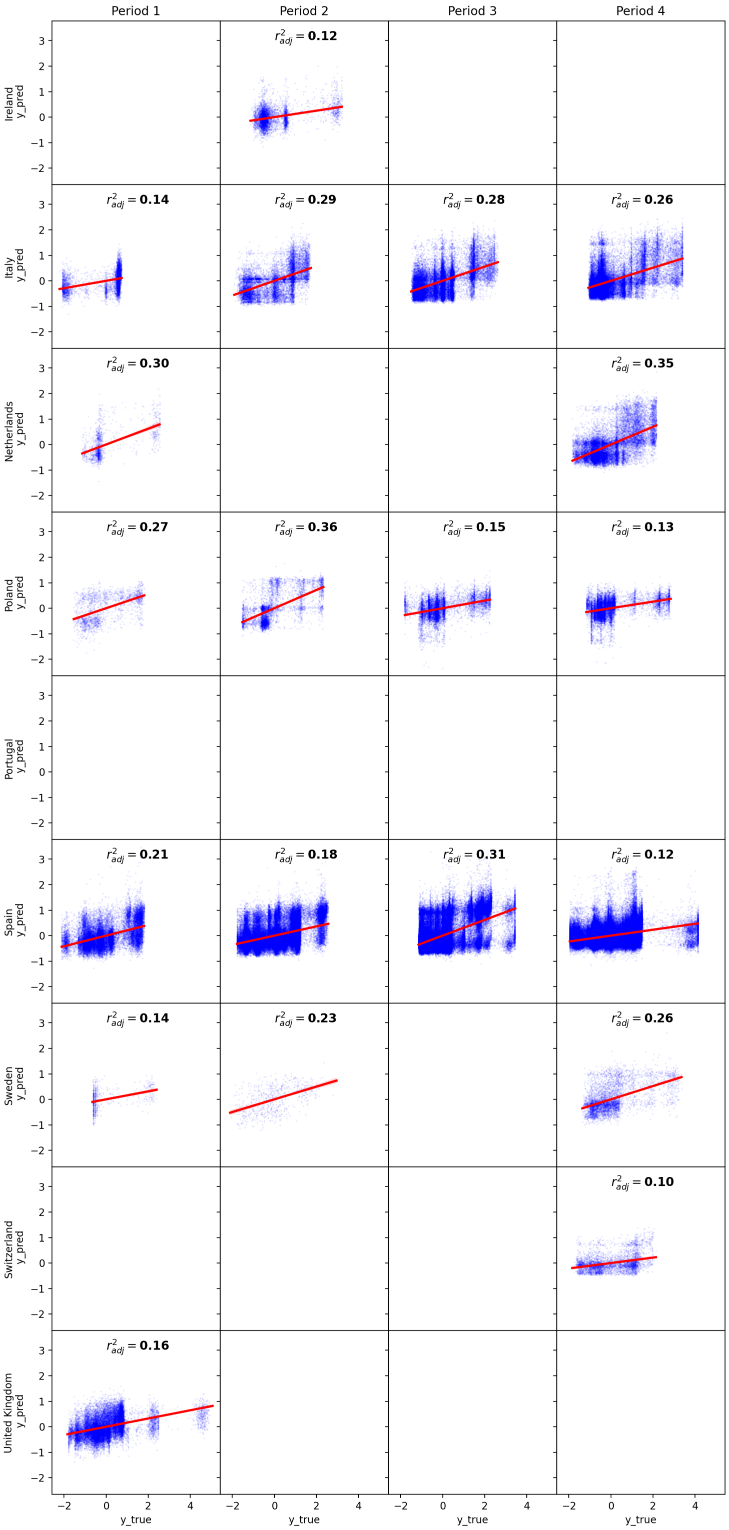

Figure 13. Regression performance modeling VHE score by parties grouped by quintiles of the left-right dimension specified by ParlGov (part 2). The x-axes represent the real target y_{true} (user's VHE score) and the y-axis the predictions from OLS models (y_{pred}).

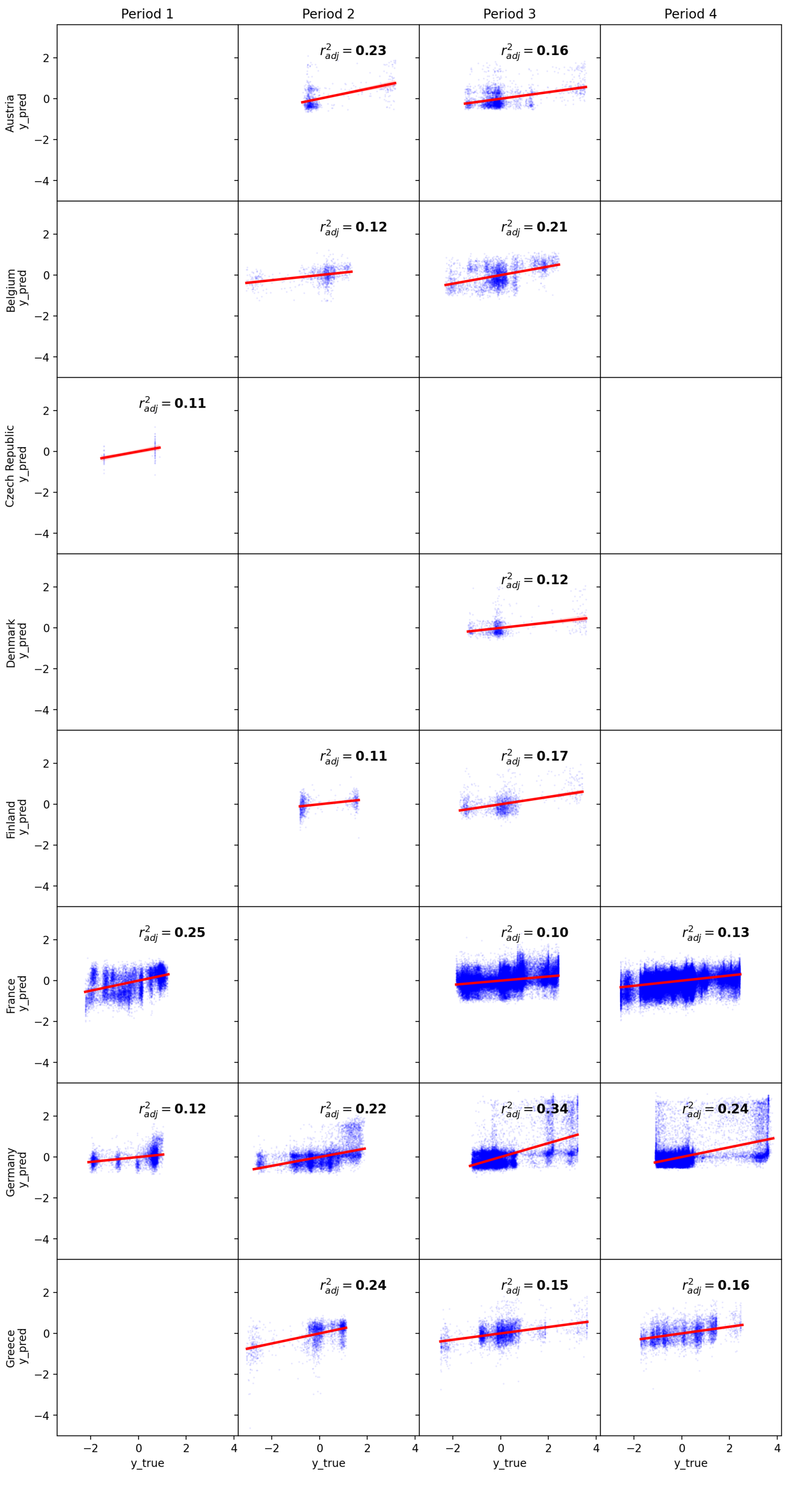

Figure 14. Regression performance modeling VHE score by parties grouped by quintiles of the liberty-authority dimension specified by ParlGov (part 1). The x-axes represent the real target y_{true} (user's VHE score) and the y-axis the predictions from OLS models (y_{pred}).

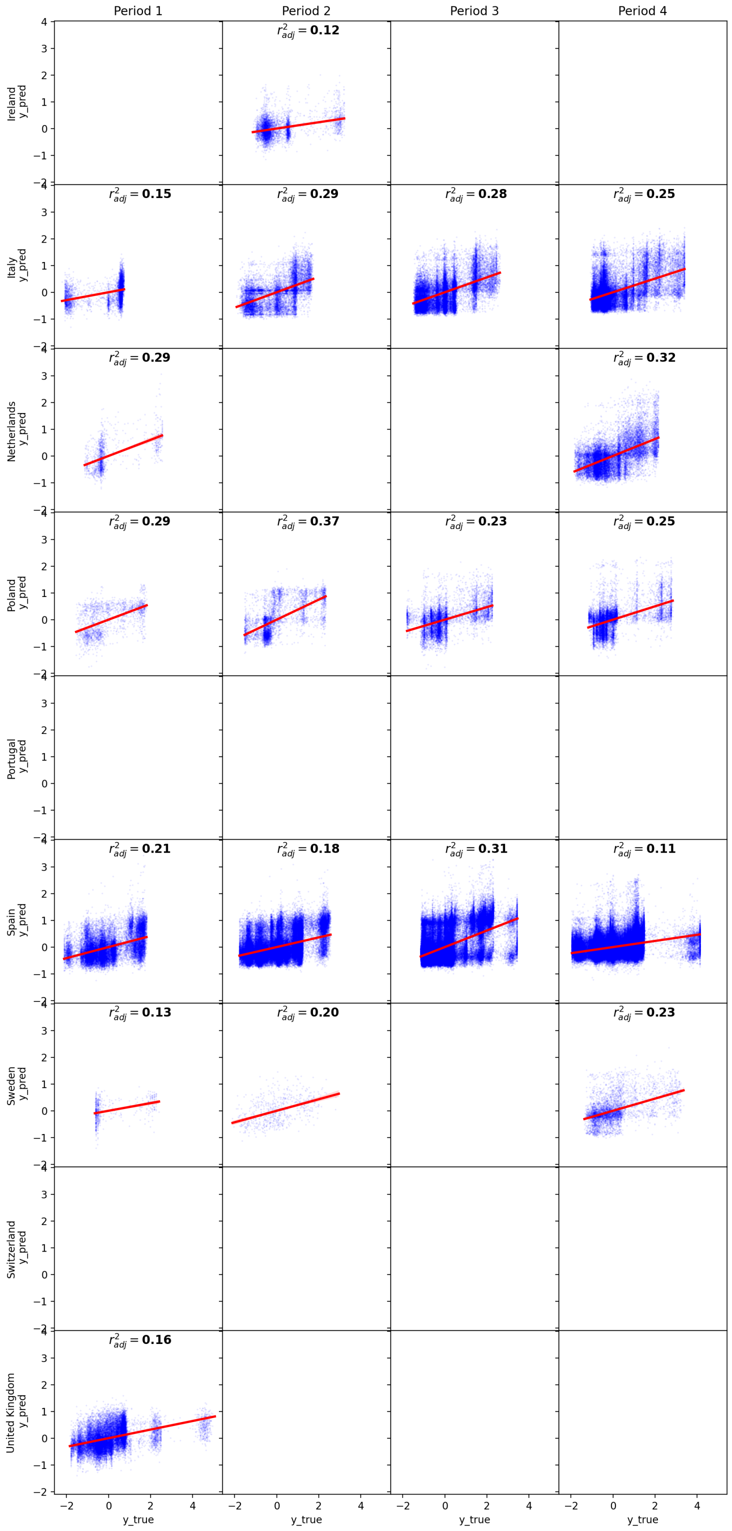

Figure 15. Regression performance modeling VHE score by parties grouped by quintiles of the liberty-authority dimension specified by ParlGov (part 2). The x-axes represent the real target y_{true} (user's VHE score) and the y-axis the predictions from OLS models (y_{pred}).

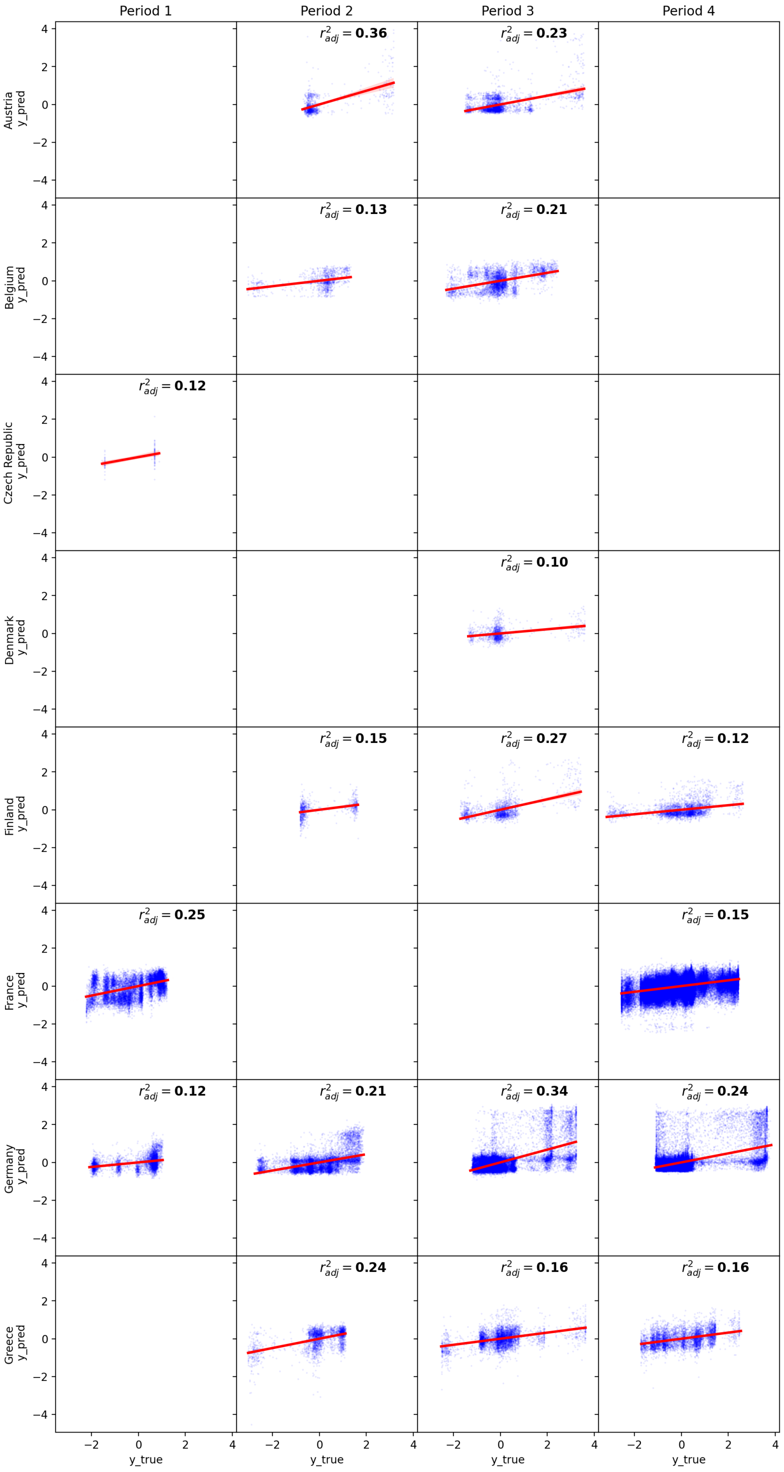

Figure 16. Regression performance modeling VHE score by parties grouped by quintiles of the anti EU - pro EU dimension specified by ParlGov (part 1). The x-axes represent the real target y_{true} (user's VHE score) and the y-axis the predictions from OLS models (y_{pred}).

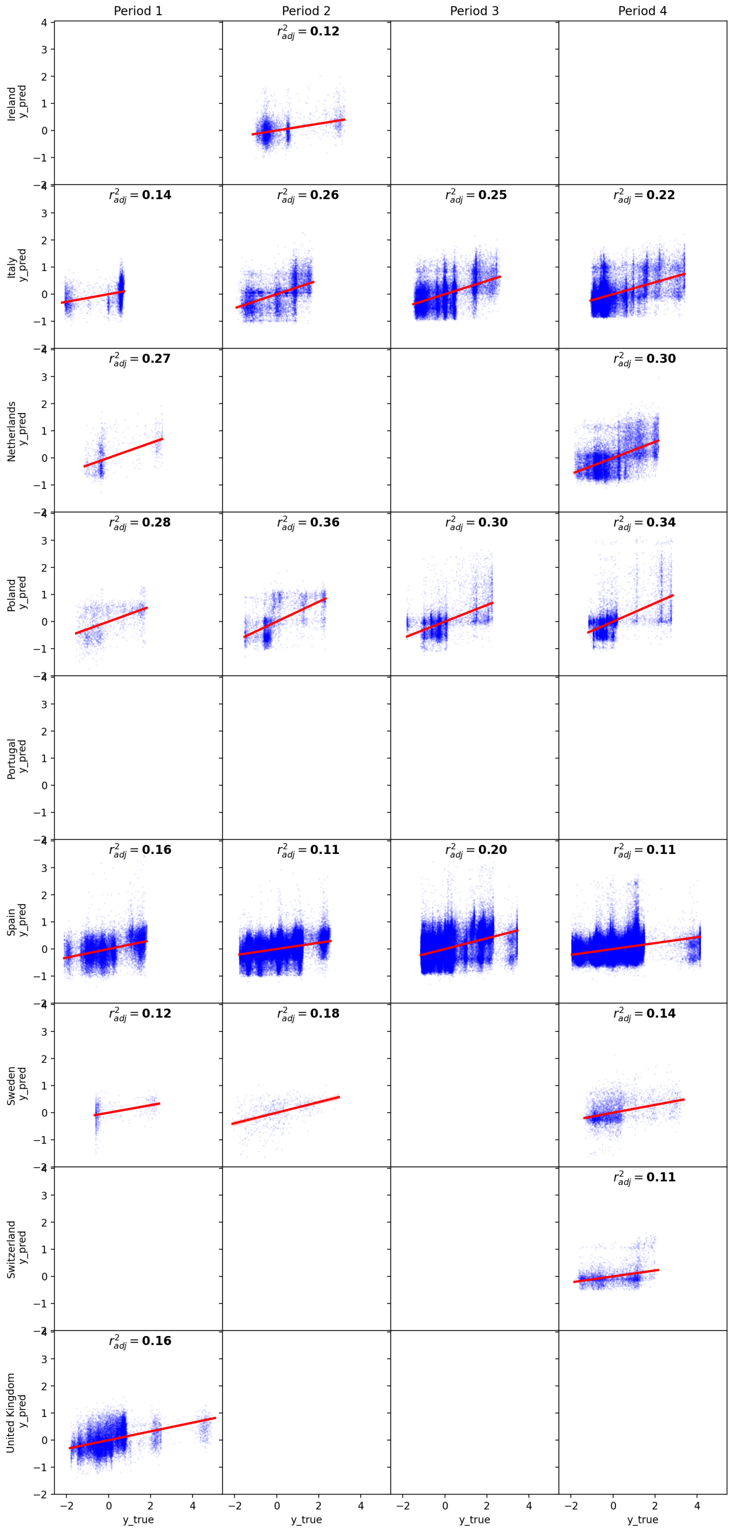

Figure 17. Regression performance modeling VHE score by parties grouped by quintiles of the anti EU - pro EU dimension specified by ParlGov (part 2). The x-axes represent the real target y_{true} (user's VHE score) and the y-axis the predictions from OLS models (y_{pred}).

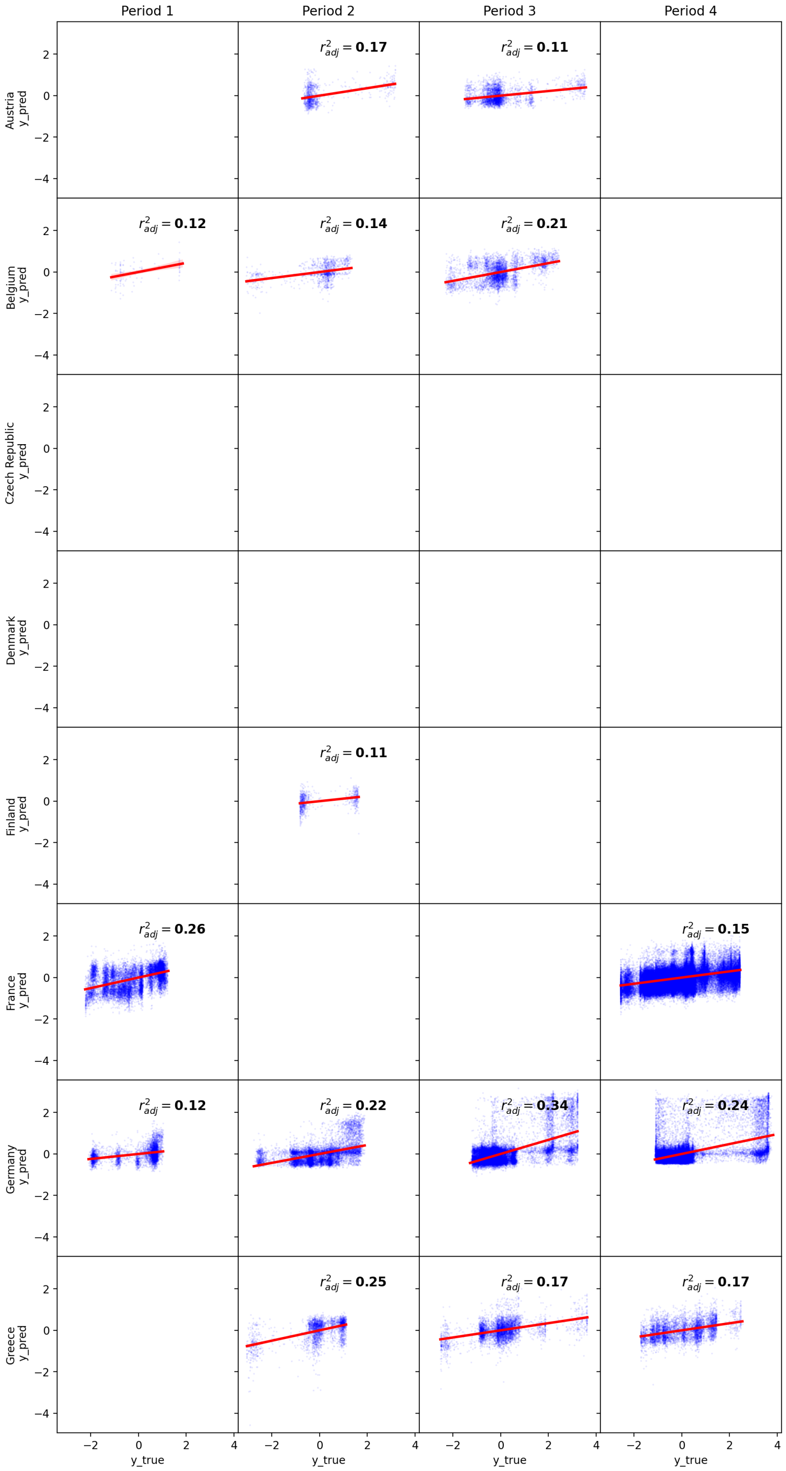

Figure 18. Regression performance modeling VHE score by parties grouped by quintiles of the state-market dimension specified by ParlGov (part 1). The x-axes represent the real target y_{true} (user's VHE score) and the y-axis the predictions from OLS models (y_{pred}).

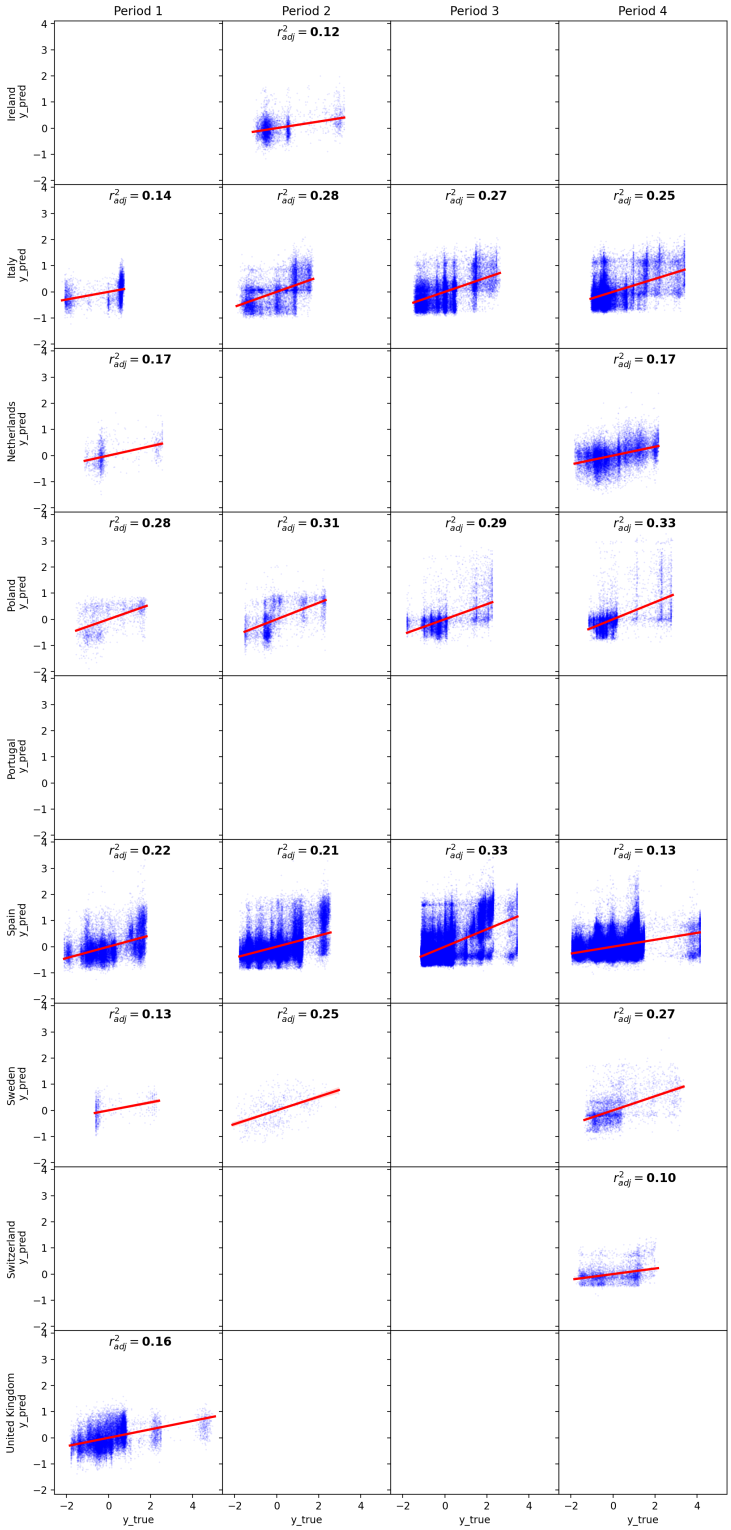

Figure 19. Regression performance modeling VHE score by parties grouped by quintiles of the state-market dimension specified by ParlGov (part 2). The x-axes represent the real target y_{true} (user's VHE score) and the y-axis the predictions from OLS models (y_{pred}).
